# Supplementary material for: GNSS multipath suppression technology based on postcorrelation and independent component analysis
Source: PLoS One. 2022 Apr 21;17(4):e0267216. doi: 10.1371/journal.pone.0267216 (PMC9022839; doi:10.1371/journal.pone.0267216)
Supplement: Attachment — Submitted filename: review.docx [file pone.0267216.s001.docx]

The paper proposes a joint post-correlation and independent component analysis method for medium and short multipath suppression. These are very mature theoretical methods. The simulation experiment gave some valid results and is short of comparison with existing methods. There is still a lot of work to be done in this manuscript and I suggest rejection. My comments are summarized bellow:

1. The multipath received in urban environment is not only the reflection signal but also diffraction, refraction etc. Please give more rigorous definition in your first paragraph of your introduction and give the reference.
2. Line 62: For “DOA”, please give the full spelling when you first introduce it.
3. Actually, there are many new methods (Neural Network, machine learning) to recognize line of sight and non-line of sight in the process of signal acquisition and tracking. You should not ignore them in your introduction.
4. Your introduction is well-written on the whole, but most of your references are too old and some references for multipath processing need to be added to the review of the multipath mitigation. In particular, the current research on the spatio-temporal correlation of multipath errors (i.e. SF, ASF, MHM, T-MHM, SD-MHM). Moreover, you should cite some references about ICA for multipath mitigation (i.e. https://link.springer.com/article/10.1007/s10291-013-0341-9) or similar to ICA method (i.e. https://link.springer.com/article/10.1007/s10291-020-01074-y). These studies should be cited but not limited to these.
5. You spend a lot of time introducing ICA. Please explain, ICA methods have two main problems. The component amplitude is not uniform and the signal source is uncertain. How did you solve or avoid? Please explain or modify in detail.
6. The experiment is too simple to simulate the Beidou navigation signals. You should add smaller multipath delay time to verify the feasibility.
7. I have no idea which generation of Beidou system the author simulated. Please give more detailed simulation parameters for your Beidou signals in table 1.
8. It is better to give a comparative experiment in Fig 3. I think most of the filtering methods can play a good role after whitening.
9. Please explain, what are the conditions for the use of the proposed method? Is it more suitable in a dynamic environment, or does it perform better in a static environment? Furthermore, it is strongly recommended to add a comparison method to reflect the advantages of the proposed method.
10. As we all know, although SNR is one of the important properties of GNSS signal, but how does the proposed method perform on PNT after suppressing multipath? The suggestion is based on a carrier to reflect the universality of the proposed method. For example, after suppressing multipath, positioning performance can be demonstrated.
