# Supplementary material for: GNSS multipath suppression technology based on postcorrelation and independent component analysis
Source: PLoS One. 2022 Apr 21;17(4):e0267216. doi: 10.1371/journal.pone.0267216 (PMC9022839; doi:10.1371/journal.pone.0267216)
Supplement: Attachment — Submitted filename: Responses to Reviewers.docx [file pone.0267216.s003.docx]

**Responses to Reviewers**

**Reviewer #2:**

***Comments 1:*** Please include the computational complexities of the proposed method and these traditional methods.

***Response:***

Thanks for your comments. The proposed method includes two parts, postcorrelation and ICA algorithm. Among them, the postcorrelation and GNSS signal tracking adopt the same algorithm. Therefore, the difference in computational complexity between the proposed method and the traditional method depends on the algorithms adopted to suppress multipath. In traditional array signal processing, the complexity of spatial smoothing is *O* (*M*^2^*L*), the complexity of MUSIC algorithm is *O* (*M*^2^*V*), and the complexity of beamforming algorithm such as LCMV is *O* (*M*^2^*L*). Where *M* is the number of array elements, *L* is the number of snapshots and *V* is the searching grid number of MUSIC algorithm. The total computational complexity of traditional methods is *O* (*M*^2^*L*+*M*^2^*V*). For the ICA algorithm used in the proposed method, only one direct signal is extracted, so the number of iterations is 1. Therefore, the computational complexity of the proposed method is *O* (*M*^2^*L*), which is smaller than the traditional method.

The description in the manuscript has been revised.

***Comments 2:*** Meanwhile, the results of the performance under different SNRs is also need included.

***Response:***

Section 4.4 shows the performance comparison between the proposed method and the traditional method under different received SNR.

**Reviewer #3:**

***Comments 1:*** This article still does some work for multipath mitigation, I hope the author can do more in this field.

***Response:***

Thank you for your previous valuable suggestions on improving this article. We will conduct more in-depth research in the field of multipath mitigation.
